# The Decadence of Opium Addiction

**Published:** 1895-12

**Authors:** Wendell Reber

**Affiliations:** Pottsville, Pa., Oculist and aurist to the Children’s Home


### THE DECADENCE OF OPIUM ADDICTION.

BY WENDELL REBER, M. D., Pottsville, Pa.,
Oculist and aurist to the Children's Home.

FROM the early history of medicine up to a few years ago, opium in its galenic or alkaloidal derivatives was the chief agent in the hands of the physician for the alleviation of all pain. Its use by the profession was world-wide, and even the laity began to look upon the drug as a convenient and comparatively safe means to have at hand for relief.

The crowded condition of many of our institutions for opium habitués today proclaims all too loudly the evils of such an indiscriminate practice, and though many a medical man has had reason to reproach himself for introducing his patient to the alluring drug, there are, even today, those who, disregarding the magnificent contributions of synthetic chemistry in the last fifteen years, cling tenaciously to papaveris as the ideal pain reliever and sleep producer.

Not so, however, with those who have patiently and studiously sought out an anodyne and hypnotic in whose train did not follow the baneful results that attend long-continued opium administration. We would not banish opium. Far from it. There are times when it becomes our refuge. But we would restrict it to its proper sphere.

In the acute stage of most inflammations, and in the closing painful phases of some few chronic disorders, it is our grandest remedy—our confidential friend. But here, the application must cease : and it is just here that the synthetic products step in to claim their share in the domain of therapy.

Among the latter, perhaps none has met with so grateful a reception as antikamnia, and justly so ; for among all the contributions of pharmaceutic chemistry none so fully merits our confidence as this one, presenting, as it does, the meritorious properties of the other synthetic antipyretics and antineuralgics, without exhibiting their depressing action on the heart.

Its range of application is wide. It is of positive value in certain forms of dysmenorrhea ; it has served me well in the pleuritic pains of advancing pneumonia, and in the arthralgies of acute rheumatism ; on several occasions I have been able to allay with it the lightning, lancinating pains of locomotor ataxia ; but nowhere do I employ it with such confidence as in the neuralgias limited to the area of distribution of the fifth nerve. Here its action is almost specific ; surpassing even the effect of aconite over this nerve.

Given a frontal-temporal-vertical or occipital neuralgia growing out of an uncorrected ocular defect, it will almost invariably arrest the head pain until such times as the ocular trouble can be corrected with glasses. In the terrific fronto-parietal neuralgia of glaucoma, or in rheumatic or post-operative iritis, it is of signal service, contributing much to the comfort of the patient, and I have sometimes thought, exerting an undeniable influence over the ocular disease. In this last group of cases I have seen the most benign effects follow the hourly administration of ten grains of antikamnia until the pain is relieved. It will seldom be necessary to exceed sixty grains of the drug.

According to the statistics of institutions for the treatment of opium addiction, there is no class of invalids from which there have been more opium habitués recruited than chronic neuralgics. If this be so, and the statement proceeds from high authority, the medical man who uses opium in chronic diseases, save as a dernier resort, is not only indulging in a reprehensible practice, but, reviewed from the standpoint of higher medical ethics, he is criminally careless in employing a dangerous drug when one that has been proven safe will meet the indications equally well.

